# Rapid molecular detection of pathogenic microorganisms and antimicrobial resistance markers in blood cultures: evaluation and utility of the next-generation FilmArray Blood Culture Identification 2 panel

**DOI:** 10.1007/s10096-021-04314-2

**Published:** 2021-08-05

**Authors:** Tanja Holma, Jukka Torvikoski, Nathalie Friberg, Annika Nevalainen, Eveliina Tarkka, Jenni Antikainen, Jari J. Martelin

**Affiliations:** HUS Diagnostic Center, HUSLAB, Department of Clinical Microbiology, University of Helsinki and Helsinki University Hospital, Helsinki, Finland

**Keywords:** Bacteremia, Blood culture, BSI, Antimicrobial resistance, Molecular detection methods, Rapid diagnostics

## Abstract

Rapid detection of pathogens causing bloodstream infections (BSI) directly from positive blood cultures is of highest importance in order to enable an adequate and timely antimicrobial therapy. In this study, the utility and performance of a recently launched next-generation fully automated test system, the Biofire FilmArray® Blood Culture Identification 2 (BCID2) panel, was evaluated using a set of 103 well-characterized microbial isolates including 29 antimicrobial resistance genes and 80 signal-positive and 23 signal-negative clinical blood culture samples. The results were compared to culture-based reference methods, MALDI-TOF, and/or 16S rDNA sequencing. Of the clinical blood culture samples, 68 were monomicrobial (85.0%) and 12 polymicrobial (15.0%). Six samples contained ESBL (*bla*_CTX-M_), two MRSA (*mec*A), and three MRSE (*mec*A) isolates. In overall, the FilmArray BCID2 panel detected well on-panel targets and resistance markers from mono- and polymicrobial samples. However, one *Klebsiella aerogenes* and one *Bacteroides ovatus* were undetected, and the assay falsely reported one *Shigella flexneri* as *Escherichia coli*. Hence, the sensitivity and specificity for detecting microbial species were 98.8% (95%CI, 95.8–99.9%) and 99.9% (95%CI, 99.8–99.9%), respectively. The sensitivity and specificity for detecting of resistance gene markers were 100%. The results were available within 70 min from signal-positive blood cultures with minimal hands-on time. In conclusion, the BCID2 test allows reliable and simplified detection of a vast variety of clinically relevant microbes causing BSI and the most common antimicrobial resistance markers present among these isolates.

## Introduction

High mortality and morbidity rates are associated with bloodstream infections (BSIs) [1, 2]. In polymicrobial BSIs, which are not uncommon (ranging from 10 to 20% of all diagnosed BSIs), the mortality rates are even higher than with monomicrobial BSIs [3–6]. Therefore, rapid administration of effective antimicrobial treatment is crucial for patient survival [7, 8]. To support this, timely and accurate identification of microorganisms are central to the optimal management of BSIs.

The “gold standard” for screening of BSIs is still blood culture followed by Gram-staining and conventional culture-based techniques. The conventional techniques have the advantage of providing accurate species identification and antimicrobial susceptibility profile but the disadvantage of requiring 1–2 additional days after signal-positive blood cultures. During the last few years, various methods have been developed to optimize the detection of the etiological agent of BSI such as fluorescence hybridization probes [9–11], pathogenic-specific PCR [12, 13], and MALDI-TOF MS [14–16]. Despite the advantages of these approaches, many of them, however, comprise multiple assay steps for the purification and extraction of the microbes and their target protein or DNA. Moreover, most of them provide limited information from polymicrobial blood culture samples. Modern automatization and advances in molecular technology, however, have recently led to the development of molecular-based high multiplex platforms, such as the FilmArray (bioMérieux, France), the ePlex (GenMark Diagnostics, USA), and the Verigene (Luminex, USA), for direct and rapid multimicrobial detection from signal-positive blood culture bottles [17–19]. These assays allow significantly shorter time to detection than conventional methods and a high performance even with polymicrobial samples, although some limitations in the first-generation assay panel content were evident (e.g., absence of the most common resistance genes from the panel) [17–19].

Here, the performance and utility of the recently launched updated version of the FilmArray Blood Culture Identification 2 panel, the BCID2 assay (bioMérieux, France), were evaluated. According to the manufacturer, the BCID2 panel identifies simultaneously 33 species/genus targets and 10 antimicrobial resistance genes. This is nine microbial targets and seven genes coding for antimicrobial resistance more than with the first-generation BCID [20] assay.

## Materials and methods

### Simulated blood culture samples

The ability of the FilmArray BCID2 panel to detect microbes from mono- and polymicrobial samples was investigated using a set of bacterial (*n* = 83) and fungal (*n* = 20) isolates originating from either ATCC or clinical blood culture specimens (Table 1). Among the bacterial isolates, 29 resistance marker genes, relevant for this study, were also included. All culture collection isolates from clinical origin had been well-identified by MALDI-TOF (Vitek MS, bioMérieux, France) and/or by 16S rDNA sequencing [21]. Samples of simulated signal-positive blood cultures were prepared by mixing 3 mL of whole blood and 8 mL of blood culture bottle medium (BacT/Alert, bioMérieux) and spiked with culture collection isolate(s) in a concentration of approximately 3 × 10^6^ CFU/ml. In total of 21 samples were prepared, of which 17 were pooled (polymicrobial), containing up to eight microbes per sample, and four contained one microbe only (Table 2).

**Table 1:** Performance of the FilmArray BCID2 panel with simulated blood culture samples containing known culture collection isolates in monomicrobial and pooled polymicrobial mixes

Isolate		Results of the BCID2 panel	
Species (*No. of isolates*)	Resistance gene marker (*No. of genes*)	Species marker (*No. of positive*)	Resistance gene marker (*No. of positive*)
*Acinetobacter baumannii* (2)	*bla*_OXA-23_ (2)^a^	*A. calcoaceticus-baumannii* complex (2)	-
*A. lwoffii* (1)	-	-	-
*Actinomyces* spp. (1)	-	-	-
*Bacillus subtilis* (1)	-	-	-
*Bacteroides fragilis* (2)	-	*B. fragilis* (2)	-
*B. ovatus* (1)	-	-^b^	-
*B. thetaiotaomicron* (1)	-	*B. fragilis* (1)^b^	-
*Candida albicans* (2)	-	*C. albicans* (2)	-
*C. auris* (2)	-	*C. auris* (2)	-
*C. dubliniensis* (1)	-	-	-
*C. glabrata* (2)	-	*C. glabrata* (2)	-
*C. krusei* (2)	-	*C. krusei* (2)	-
*C. parapsilosis* (2)	-	*C. parapsilosis* (2)	-
*C. tropicalis* (2)	-	*C. tropicalis* (2)	-
*Clostridium clostridioforme* (1)	-	-	-
*Corynebacterium amycolatum* (1)	-	-	-
*C. pseudodiphtheriticum* (1)	-	-	-
*Cryptococcus gattii* (3)	-	*C. neoformans/gattii* (3)	-
*C. neoformans* (3)	-	*C. neoformans/gattii* (3)	-
*Enterobacter cloacae* (2)	-	*E. cloacae* complex (2)	-
*Enterococcus faecalis* (3)	*vanB* (3)	*E. faecalis* (3)	*vanA/B* (3)
*Enterococcus faecium* (2)	*vanA* (2)	*E. faecium* (2)	*vanA/B* (2)
*Escherichia coli* (6)	*bla*_OXA-48_ (2), *bla*_CTX-M_ (2), *mcr*-1 (2), *bla*_OXA-181_ (1)	*E. coli* (6)	OXA-48-like (3)^c^, CTX-M (2), and *mcr*-1 (2)
*Haemophilus influenzae* (2)	-	*H. influenzae* (1)	-
*H. parainfluenzae* (1)	-	-	-
*Klebsiella aerogenes* (2)	-	*K. aerogenes* (1)^d^	-
*K. oxytoca* (2)	-	*K. oxytoca* (2)	-
*K. pneumoniae* (6)	*bla*_KPC_ (2), *bla*_VIM_ (2), *bla*_NDM_ (2)	*K. pneumoniae* group (6)	KPC (2), VIM (2), and NDM (2)
*Lactobacillus spp.* (1)	-	-	-
*Listeria monocytogenes* (3)	-	*L. monocytogenes* (3)	-
*Neisseria meningitidis*, serogroup A (2) and serogroup B (1)	-	*N. meningitidis* (encapsulated) (3)	-
*N. mucosa/sicca*	-	-	-
*Proteus mirabilis* (2)	-	*Proteus* spp. (2)	-
*Pseudomonas aeruginosa* (4)	*bla*_IMP_ (2)	*P. aeruginosa* (4)	IMP (2)
*Saccharomyces cerevisiae* (1)	-	-	-
*Salmonella enterica* spp. *enterica* serovar Enteritidis (1)	-	*Salmonella* spp. (1)	-
*S. enterica* spp. *enterica* serovar Typhi (1)	-	*Salmonella* spp. (1)	-
*S. enterica* spp. *enterica* serovar Typhimurium (2)	-	*Salmonella* spp. (2)	-
*Serratia marcescens* (2)	-	*S. marcescens* (2)	-
*Shigella flexneri* (1)	-	*E. coli* (1)	-
*Staphylococcus aureus* (6)	*mecA* (6)	S. aureus (6)	*mecA/C* and MREJ (MRSA) (6)
*S. epidermidis* (3)	*mecA* (1)	*S. epidermidis* (3)	*mecA/C* (1)
*S. hominis* (1)	-	*Staphylococcus* spp. (1)	-
*S. lugdunensis* (2)	-	*S. lugdunensis* (2)	-
*Stenotrophomonas maltophilia* (2)	-	*S. maltophilia* (2)	-
*Streptococcus agalactiae* (2)	-	*S. agalactiae* (group B) (2)	-
*S. dysgalactiae* spp. *equisimilis* group C (1) and group G (1)	-	*Streptococcus* spp. (2)	-
*S. mitis* (1)	-	*Streptococcus* spp. (1)	-
*S. pneumoniae* (2)	-	*S. pneumoniae* (2)	-
*S. pyogenes* (2)	-	*S. pyogenes* (group A) (2)	-

-, negative/no detection

MREJ, SCCmec right-extremity junction

^a^The *bla*_OXA-23_ detection is not included into the FilmArray BCID2 panel

^b^The species classified as a member of *B. fragilis* group

^c^The *bla*_OXA-181_ gene belongs to a group of OXA-48-like carbapenem-hydrolyzing class D β-lactamases

^d^One *K. aerogenes* isolate was undetected with the FilmArray BCID2 panel. The isolate was confirmed by 16S rDNA sequencing as *K. aerogenes*

**Table 2:** Number of microbes and resistance markers in simulated blood culture sample pools used for the FilmArray BCID2 panel testing

No of targets per sample	Samples																				
	S1	S2	S3	S4	S5	S6	S7	S8	S9	S10	S11	S12	S13	S14	S15	S16	S17	S18	S19	S20	S21
*Gram-negative organisms*	1	3	5	5	3	1	1	1	2	1	1	1	1	1	4	1	3	5	4	3	1
*Gram-positive organisms*	5	2	1	0	1	2	3	3	3	0	0	3	4	0	0	5	2	1	0	1	0
*Yeasts*	1	2	2	1	2	1	1	1	0	0	0	0	1	0	0	1	2	2	1	2	0
*Resistance markers*	4	0	1	2	3	0	1	1	1	0	0	1	2	1	1	4	0	1	2	3	1
Total	11	7	8	8	9	4	6	6	6	1	1	5	8	2	5	11	7	9	7	9	2
No. of correct detection with the FilmArray BCID2 panel	11	7	8	7^a^	9	3^b^	6	6	6	0^c^	1	5	8	2	4^a^^,b^	11	7	9	6^a^	9	2

^a^One *K. aerogenes* isolate was not detected with the FilmArray BCID2 panel in sample S4. The isolates was added and reanalyzed in samples S15 and S19. No detection was observed in any of the samples

^b^*B. ovatus*, a member of *B. fragilis* group in sample S6, was not detected as *B. fragilis*, while *B. thetaiotaomicron*, another member of *B. fragilis* group, in S15 was detected as *B. fragilis*

^c^*S. flexneri* in sample S10 was incorrectly identified as *E. coli*

### Clinical samples

The clinical blood culture samples were collected at the region of HUS hospital district and analyzed at HUS Diagnostic Center, Department of Clinical Microbiology (HUSLAB, Finland). In HUSLAB, approximately 140 000 blood culture specimens are screened each year, of which approximately 7% are growth-positive. E. coli and S. aureus among few other common bacteria causing BSI form vast majority of strains isolated from positive blood cultures samples. Testing consecutive samples in this setting would yield, with high probability, a one-sided view of the test in hand given the limited amount of test kits assigned for the study. To overcome these limitations and to simulate situations where the results would yield maximal clinical impact following criteria for the samples to be tested were made. A single positive blood culture bottle per patient would be tested if a) small gram-positive rod, gram-negative cocci, yeast, or two or more findings would be identified in gram stain; and/or b) the patient was known to be a carrier of ESBL producing Enterobacteriaceae and/or MRSA. In addition, to provide a larger spectrum of causative agents to be tested and to ensure that all of the test kits assigned for the study would be used in limited time some of the samples were selected by authors guided by the principle to provide a diverse sample material for the study. A total of 103 blood culture bottles (BacT/Alert, bioMérieux, France), one bottle per patient, confirmed to contain either Gram-negative bacteria, Gram-positive rods, Gram-positive diplococci, cocci in chains, yeast, or polymicrobial growth by Gram-staining and evaluation by a clinical microbiologist, were analyzed between November 2020 and January 2021 with the FilmArray BCID2 assay and routine reference methods. Routine methods included subculture from the signal-positive bottle on rich universal agars, such as blood, chocolate, and fastidious anaerobe agar plates with incubation at 35 °C in normal atmosphere as well as in elevated CO_2_ and in anaerobic conditions for 24 to 48 h. The short-incubation MALDI-TOF (si-MALDI-TOF) technique [21] was used for the preliminary identification of microbial growth from the secondary cultures after 6 hours of incubation. The identifications were confirmed by repeating the MALDI-TOF (Vitek MS, bioMérieux, France) analysis after 24 or 48 hours of incubation from distinct colonies. In case of unclear results, the confirmation was done by 16S rDNA sequencing [21]. Antimicrobial susceptibilities were determined using disc (Oxoid, UK) and/or MIC-gradient (M.I.C.E, Oxoid, Basingstoke, UK and ETest, bioMérieux, France) diffusion tests on Mueller-Hinton agar (BD, USA) at 35 °C for 24 to 48 hours. Phenotypic susceptibility profile for each isolate was interpreted according to the EUCAST standard [22]. Genotypic confirmation was done in national reference laboratory (Finnish Institute for Health and Welfare).

Sample runs with the FilmArray BCID2 assay were performed according to the manufacturer’s instructions. Performance of the BCID2 panel was calculated using MedCalc statistical analysis software (MedCalc Software Ltd, Belgium). The 95% confidence intervals (95% CI) were approximated using the Clopper-Pearson method, i.e., binomial exact confidence interval method.

The Fisher’s exact test was used to determine statistical significance of the differences among the BCID2 panel and routinely used reference methods in species and resistance marker identification within specified time period (within 8 hours from signal of positivity).

## Results

From the simulated sample mixes, the BCID2 panel provided a correct identification in 99.9% (95% CI, 99.7–100%) of all target microbes and in 100% (95% CI, 98.7–100%) of all resistance marker genes (Table 1). The false detections consisted of one *Klebsiella aerogenes* isolate, which was repeatedly undetected from different sample mixes and *Shigella flexneri*, which was misidentified as *Escherichia coli*. In addition, *Bacteroides ovatus*, a member of *B. fragilis* group, was not detected as *B. fragilis*, whereas *B. thetaiotaomicron*, also a member of *B. fragilis* group, was detected as *B. fragilis*. This was considered as a false-negative detection for the *B. ovatus* isolate. In general, the BCID2 panel showed excellent performance from the multipositive samples providing correct identification even from samples containing 11 different targets.

Of the 103 clinical blood culture samples, 80 were signal-positive containing either Gram-negative bacteria, Gram-positive rods, diplococci, cocci in chains, yeasts, or polymicrobial growth by Gram-staining. The proportion of polymicrobial samples was 15.0% (*n* = 12). The remaining 23 samples, included in the study, were signal- and growth-negative. In total, 36 Gram-positive bacteria, consisting of 12 different species, 36 Gram-negative bacteria, consisting of nine different species, and five yeasts, consisting of two different species, were correctly identified by the BCID2 panel (Table 3). The most common microbes recovered were *E. coli* (*n* = 15), *Streptococcus* spp. (*n* = 11), *Klebsiella* spp. (*n* = 10), and *L. monocytogenes* (*n* = 6). The BCID2 panel covered 81.9% of the microbes found from the blood culture samples. The missing (off-panel) species were mainly anaerobic Gram-negative rods and aerobic Gram-positive rods other than *Listeria* spp. One unresolved discordant result was observed from a sample containing *Enterococcus avium*, *Pseudomonas aeruginosa*, and *E. coli* from where the BCID2 panel yielded *Streptococcus* spp., *P. aeruginosa*, and *E. coli* positive result. Growth appropriate for streptococci was not observed on any agar plates nor was the isolate confirmed by any other method. Along with the Gram-negative and Gram-positive bacteria, 10 resistance genes (six *bla*_CTX-M_ and four *mecA/C* genes) were detected and correctly identified by the BCID2 panel (Table 3). No additional resistance markers were found by the routine reference methods. Thus, the overall sensitivity and specificity of the BCID2 panel from the clinical sample set were 100% (95% CI, 95.9–100%) and 99.9% (95% CI, 99.8–100%), respectively.

**Table 3:** Results of the FilmArray BCID2 assay from clinical blood culture samples (*n* = 103, one per patient)

Performance of the FilmArray BCID 2 panel						
The BCID2 panel target organisms and resistance markers	TP	TN	FN	FP	Sensitivity (95% CI)	Specificity (95% CI)
Gram-positive bacteria						
*Enterococcus faecalis*	0	103	0	0	N/A	100% (96.5–100%)
*Enterococcus faecium*	0	103	0	0	N/A	100% (96.5–100%)
*Listeria monocytogenes*	6	97	0	0	100% (54.1–100%)	100% (96.3–100%)
*Staphylococcus spp.*	1^a^	102	0	0	100% (2.5–100%)	100% (96.5–100%)
*Staphylococcus aureus*	5	98	0	0	100% (47.8–100%)	100% (96.3–100%)
*Staphylococcus epidermidis*	4	99	0	0	100% (39.8–100%)	100% (96.3–100%)
*Staphylococcus lugdunensis*	1	102	0	0	100% (2.5–100%)	100% (96.5–100%)
*Streptococcus spp.*	11^b^	91	0	1^c^	100% (71.5–100%)	98.9% (94.1–100%)
*Streptococcus agalactiae* (group B)	4	99	0	0	100% (39.8–100%)	100% (96.3–100%)
*Streptococcus pneumoniae*	2	101	0	0	100% (15.8–100%)	100% (96.4–100%)
*Streptococcus pyogenes* (group A)	2	101	0	0	100% (15.8–100%)	100% (96.4–100%)
Gram-negative bacteria						
*Acinetobacter calcoaceticus-baumannii* complex	1	102	0	0	100% (2.5–100%)	100% (96.5–100%)
*Bacteroides fragilis*	1	102	0	0	100% (2.5–100%)	100% (96.5–100%)
*Enterobacterales*	2^d^	101	0	0	100% (15.8–100%)	100% (96.4–100%)
*Enterobacter cloacae* complex	0	103	0	0	N/A	100% (96.5–100%)
*Escherichia coli*	15	88	0	0	100% (78.2–100%)	100% (95.9–100%)
*Klebsiella aerogenes*	0	103	0	0	N/A	100% (96.5–100%)
*Klebsiella oxytoca*	5	98	0	0	100% (47.8–100%)	100% (96.3–100%)
*Klebsiella pneumoniae* group	5	98	0	0	100% (47.8–100%)	100% (96.3–100%)
*Proteus spp.*	1	102	0	0	100% (2.5–100%)	100% (96.5–100%)
*Salmonella spp.*	0	103	0	0	N/A	100% (96.5–100%)
*Serratia marcescens*	0	103	0	0	N/A	100% (96.5–100%)
*Haemophilus influenzae*	1	102	0	0	100% (2.5–100%)	100% (96.5–100%)
*Neisseria meningitidis*	0	103	0	0	N/A	100% (96.5–100%)
*Pseudomonas aeruginosa*	5	98	0	0	100% (47.8–100%)	100% (96.3–100%)
*Stenotrophomonas maltophilia*	0	103	0	0	N/A	100% (96.5–100%)
Yeasts						
*Candida albicans*	3	100	0	0	100% (29.2–100%)	100% (96.4–100%)
*Candida auris*	0	103	0	0	N/A	100% (96.5–100%)
*Candida glabrata*	2	101	0	0	100% (15.8–100%)	100% (96.4–100%)
*Candida krusei*	0	103	0	0	N/A	100% (96.5–100%)
*Candida parapsilosis*	0	103	0	0	N/A	100% (96.5–100%)
*Candida tropical*	0	103	0	0	N/A	100% (96.5–100%)
*Cryptococcus neoformans/gattii*	0	103	0	0	N/A	100% (96.5–100%)
Resistance markers						
CTX-M	6	97	0	0	100% (54.1–100%)	100% (96.3–100%)
IMP	0	103	0	0	N/A	100% (96.5–100%)
KPC	0	103	0	0	N/A	100% (96.5–100%)
NDM	0	103	0	0	N/A	100% (96.5–100%)
OXA-48-like	0	103	0	0	N/A	100% (96.5–100%)
VIM	0	103	0	0	N/A	100% (96.5–100%)
mecA/C	1	102	0	0	100% (2.5–100%)	100% (96.5–100%)
mecA/C and MREJ (MRSA)	3	100	0	0	100% (29.2–100%)	100% (96.4–100%)
mcr-1	0	103	0	0	N/A	100% (96.5–100%)
vanA/B	0	103	0	0	N/A	100% (96.5–100%)

^a^One sample contained *Staphylococcus hominis*

^b^Of the 11 samples, two contained *Streptococcus anginosus*, one *S. intermedius*, four *S. viridans* group, and four *S. dysgalactiae* spp. *equisimilis*

^c^The BCID2 panel yielded *Streptococcus* spp. positive result from a sample containing a mixed growth of *E. avium*, *P. aeruginosa*, and *E. coli*. The result could not be confirmed with reference methods and was considered as false-positive detection

^d^Of the two samples detected as Enterobacterales with the BCID2 panel, one contained *Raoultella ornithinolytica* and the other one *Serratia rubidaea*

Species identification with the routine culture-based methods was available for 68.0% of the samples within 24–48 h, when short-incubation (si-)MALDI-TOF could not be utilized (e.g., with polymicrobial growth and slowly growing organism), and for 32.0% of the samples within 6–8 h, when si-MALDI-TOF could be utilized. For antimicrobial susceptibilities, the total turnaround time was 24–48 h with the routine methods. The direct analysis of the clinical blood culture samples with the automated BCID2 panel enabled species identification and detection of the most common antimicrobial resistance genes within 70 min. This provided significant (*P* value < 0.0001) time reduction as compared with the routine methods.

The BCID2 panel invalidity rate from the clinical blood culture bottle samples was 1.0% (1/103).

## Discussion

In the current study, the performance and usability of the FilmArray BCID2 panel for rapid detection of organism from blood culture samples were evaluated. The new BCID2 panel has been improved greatly as it detects nine microbial targets and seven resistance genes more than the first-generation BCID panel [20, 23]. In addition, the assay run time has been reduced from 90 to 70 min. Hence, the panel provides a detection of 33 most common microbial species/genus causing BSI and 10 antimicrobial resistance marker genes reducing the time to species identification and detection of major resistance genes by at least a full day as compared with conventional culture-based methods.

The performance of the BCID2 panel was excellent providing overall 100% sensitivity and 100% specificity for on-panel antimicrobial resistance markers and 98.8% sensitivity and 99.9% specificity for on-panel microbial targets. Of the four cases from which the BCID2 panel failed to correctly identify the isolates, two contained off-panel organisms (*E. avium* and *S. flexneri*) according to reference methods. Of these, *S. flexneri* in monomicrobial growth was falsely identified as *E. coli* which is a severe misidentification, due to the low incidence but potentially life-threatening outcomes of *S. flexneri* bacteremia. The misidentification may occur if, e.g., β-glucuronidase gene (uidA), which is present in both species, is used for *E. coli* detection [24, 25]. Although these two organisms might be difficult to distinguish, there are genes that could be used for accurate differentiation and species detection [24]. Here, the *S. flexneri* isolate was used in simulated sample and from pure culture to ensure the result reliability.

The sample containing *E. avium*, however, remained unresolved as it contained polymicrobial growth of *E. avium*, *P. aeruginosa*, and *E. coli* of which *P. aeruginosa* and *E. coli* were correctly identified. With the reference methods, the reported *Streptococcus* spp. result could not be confirmed and was thus considered as a false detection from the *E. avium* isolate. However, since the sample contained polymicrobial growth, it is possible that streptococci, which could have been overgrown by the other species, was present in the sample but not detected by the culture-based reference methods. Sadly, the *Enterococcus* genus level detection, which was included in the first-generation BCID panel, has been removed from the BCID2 panel. It could have been used for the differentiation of *Enterococcus* spp. and *Streptococcus* spp. and used here to conclude whether or not the sample contained all four species (*P. aeruginosa*, *E. coli*, *Enterococcus* spp., and *Streptococcus* spp.). The differentiation of enterococci and streptococci genus from BSI samples is important, in general, due to their different natural resistance traits and thus different treatment options.

Of the other two samples yielding false-negative results with the BCID2 panel, one contained *K. aerogenes* (on-panel target) isolate which was confirmed as *K. aerogenes* by MALDI-TOF and 16S rDNA sequencing and the other one *B. ovatus* which is a member of the *B. fragilis* group. The BCID2 panel yielded no result from the *B. ovatus* while providing *B. fragilis* positive results from a sample containing *B. thetaiotaomicron*, another member of the *B. fragilis* group. All of these species are abundant in colon and important pathogens in polymicrobial infections [26]. Moreover, according to Brook, *B. thetaiotaomicron* and *B. ovatus* covers approximately 21% of all infections caused by the *B. fragilis* group and are associated with high mortality rate (20–30%) when inducing bacteremia [26].

Regarding the above-mentioned discrepancies it is interesting to note that three of the four misidentifications observed in this study are related to targets introduced in the panel of the new version of the kit. Consequently, further evaluation with more isolates within this group of microbes would be highly beneficial.  

In addition to the FilmArray BCID2 panel, there are only few other fully automated assays that provides a broad spectrum of species identification and detection of antimicrobial resistance genes directly from signal-positive blood culture bottles. Of these, the ePlex® Blood Culture Identification (BCID) panel (Genmark Diagnostics, USA) can detect multiple microbes and resistance genes (i.e., *bla*_KPC_, *bla*_NDM_, *bla*_VIM_, *bla*_IMP_, and *bla*_OXA-48-like_ genes in BCID-GN panel) within 90 min but is limited to detect only one type of organisms with a one type of test panel (e.g., BCID-GN for Gram-negative bacteria and BCID-GP for Gram-positive bacteria), and none of the panels are yet CE-IVD marked [27]. The Verigene blood culture assay (Luminex, USA) has the same limitation with test panels than the ePlex and requires approximately 2.5 h of run time per sample [28].

The limitation of this study was the low number of positive genes coding for carbapenem and vancomycin resistance in clinical samples, due to the low prevalence of carbapenem-producing organisms and vancomycin-resistant enterococci in BSI in Finland [29–31].

In conclusion, our results show that the FilmArray BCID2 panel proved to be a well-performing and invaluable tool for the early detection of common clinical isolates causing BSI and their antibiotic resistance genes while providing rapid aid for clinicians and helping them to administer effective antimicrobial therapy more hastily and therefore reducing patient mortality [32]. Whether to use FilmArray BCID2 panel to test all positive blood culture bottles regardless of the gram stain result or not depends on multiple factors. In an ideal situation where the most accurate and timely microbiological diagnosis is paramount FilmArray BCID2 panel could be used to test all positive blood culture bottles. However, this kind of approach can easily lead to excessive overall costs and increase the laboratory workload unnecessarily. A more frugal manner of testing only one blood culture bottle per patient might be a recommendable approach for routine diagnostic practices in most laboratories. In surroundings where resources are scarce, or the capacity of the test system is limited a more controlled approach for FilmArray BCID2 panel use could be implemented focusing on the cases for which the test provides maximal clinical impact. Before implementing this kind of diagnostic test to the routine practices, we encourage to assess the need for the test and the proposed use for the test locally with infectious disease specialists to integrate the new diagnostic means to local antimicrobial stewardship guides and to maximize the benefits of the test without increasing the overall costs of diagnostics unnecessarily. However, the overall costs of diagnostics would increase with the FilmArray BCID2 panel as compared with the conventional methods.

## Data Availability

All relevant data are made available in the manuscript.
